# Head and Neck Cancer Immunotherapy: Molecular Biological Aspects of Preclinical and Clinical Research

**DOI:** 10.3390/cancers15030852

**Published:** 2023-01-30

**Authors:** Rajdeep Chakraborty, Charbel Darido, Fei Liu, Maciej Maselko, Shoba Ranganathan

**Affiliations:** 1 Applied Biosciences, Faculty of Science and Engineering, Macquarie University, Sydney, NSW 2109, Australia; 2 Peter MacCallum Cancer Centre, Melbourne, VIC 3000, Australia; 3 Sir Peter MacCallum Department of Oncology, The University of Melbourne, Parkville, VIC 3010, Australia; 4 School of Natural Sciences, Faculty of Science and Engineering, Macquarie University, Sydney, NSW 2109, Australia

**Keywords:** cancer, immunotherapy, head and neck squamous cell carcinoma, T cell, vaccines, interleukins, adoptive T-cell transfer, tumour-infiltrating lymphocytes, chimeric antigen receptor, immune check point therapy

## Abstract

**Simple Summary:**

Immune therapies are the most recent advancements in the field of cancer and particularly in head and neck cancer. Cancer spread is attributed to the failure of immune cells to kill the tumour. Cancer patients either lack immune cells or the immune cells are restricted by cancer mechanisms attacking them. Immunotherapy boosts the patient’s immune system to fight the spread of tumours. In some treatment protocols, immune cells are directly transfused to patients, and in some cases, cancer mechanisms that restrict the immune cells from killing tumours are inhibited. In this review, we attempted to exhibit the biology of immune therapy and highlight recent clinical success in the field of head and neck cancer immunotherapy.

**Abstract:**

Breakthrough research in the field of immune checkpoint inhibitors and the development of a human papilloma virus vaccine triggered a plethora of research in the field of cancer immunotherapy. Both had significant effects on the treatment of head and neck squamous cell carcinoma. The advent of preclinical models and multidisciplinary approaches including bioinformatics, genetic engineering, clinical oncology, and immunology helped in the development of tumour-infiltrating lymphocytes (TILs) and chimeric antigen receptor (CAR) T-cell therapy. Here, we discuss different immunotherapies such as adoptive T-cell transfer, immune checkpoint inhibitors, interleukins, and cancer vaccines for the treatment of head and neck cancer. This review showcases the intrinsic relation between the understanding and implementation of basic biology and clinical practice. We also address potential limitations of each immunotherapy approach and the advantages of personalized immunotherapy. Overall, the aim of this review is to encourage further research in the field of immunotherapy for head and neck cancer.

## 1. Introduction

The twentieth century was the era of enlightenment for the world of cancer immunobiology. The 2018 Nobel Prize winners Allison and Honjo extensively worked on immune checkpoint inhibitors. Their research opened up a plethora of projects focussing primarily on T-cell immune checkpoint molecules cytotoxic T lymphocyte antigen 4 (CTLA4) and programmed cell death 1 (PD1) [1]. Advances in tumour biology demonstrate the tight interplay between the immune system and healthy and transformed neoplastic cells. The advent of novel models of disease to test the role of immune surveillance in restricting cancer progression have escalated immunobiology research [2]. Immunoinformatics approaches have led to the prediction of MHC-binding peptides [3], laying the foundation for the development of personalized immunotherapy and cancer vaccines by predicting candidate epitopes that are capable of precise cellular and humoral responses.

The hallmarks of cancer conceptualization proposed by Hanahan and Weinberg summarize the complexity of cancer phenotypes and genotypes into a generalized set of underlying principles. Immune evasion is one of their emerging hallmarks of cancer [2]. Immunocompromised individuals exhibited a striking increase in certain cancers, validating the hypothesis that a defective immune system encourages the growth of cancers [2]. The hypothesis was validated was by genetically engineering preclinical cancer mouse models to be immunocompromised, with subsequent assessment for the development of carcinogen-induced tumours. The immunocompromised mice showed higher tumour burden compared to an immunocompetent mouse model [2]. Immune family members such as CD8^+^ cytotoxic T lymphocytes (CTLs), CD4^+^ T_H_1 helper T cells, and natural killer (NK) cells, were pinpointed when abrogation of their function or their lack of development caused appreciable increase in the incidences of neoplasms. Additionally, mice models were more susceptible to tumour growth when both lymphocyte functions were abrogated [2]. Thus, it was evident that both innate and adaptive arms of the immune system are essential to eradicate neoplastic transformed cells [2]. Additionally, syngeneic immunocompetent mice showed greater resistance to the development of secondary tumours compared to immunocompromised mice when primary tumours from both types of hosts were transplanted to the other [4]. Evidence from clinical epidemiology further emphasises the notion of antitumour immunity [5]. Notable examples from ovarian and colon cancer tissue samples showing heavy CTL and NK cell burden show better prognosis compared to colon cancer patients with less lymphocyte infiltration [6]. Serendipitously, it has also been found that cancer cells secrete certain immunosuppressive proteins and, through subtle mechanisms, recruit immunosuppressive regulatory T cells and myeloid-derived suppressor cells that can result in immune evasion. Overall, the activity of immune molecules is an imperative mechanism counteracting the progression of cancer.

In this review, we summarize different approaches to immunotherapy, namely adoptive T-cell transfer (ATC), immune checkpoint blockade, and cancer vaccines, and preview future research based on interleukin biology to mitigate the harmful effects of cancer progression, particularly of the head and neck. Lastly, we address emerging immune biology preclinical models to elucidate the role of immune molecules in cancer.

## 2. Adoptive T-Cell Transfer

ATC therapy involves infusing autologous and allogenic T cells into cancer patients [7]. This therapy can use either tumour-infiltrating lymphocytes (TILs) or chimeric antigen receptor (CAR) T cells. The U.S. National Cancer Institute pioneered TIL therapy in metastatic melanoma [8]. Lymphocytes sorted from cancer biopsy samples were expanded by interleukin 2 (IL-2) treatment and reinfused into the patient with subcutaneous IL-2 [9], resulting in an initial outcome of 34% efficacy, with a median response of 4 months [9]. A follow-up study adopting a similar protocol on 93 patients reported a complete regression of tumours in approximately 20% of patients, 95% of whom were disease-free for the next three years [10]. Acquisition of higher neoantigen-specific TIL through a modern high-throughput sorting procedure resulted in better outcomes in metastatic breast cancer patients [11]. Additionally, knocking down the negative regulator of T cell receptor (TCR) signalling in murine models resulted in amplification of ATC therapy [12]. The major roadblock of the TIL approach is the acquisition of high antigen-specific effector T cells [13]. Ostensibly most of the cells are regulatory T cells that downregulate the immune response towards the cancer cells. This led to the development of engineered lymphocytes for ATC. 

However, the inherent limitation of the engineered TCR is that it is restricted to the tumour antigens presented by the major histocompatibility complex (MHC) rather than surface antigens on neoplastic cells [14].

The advent of synthetic CARs opened new possibilities that bypass MHC restriction and channelize specific target molecules on the surface of the tumour cells [15]. CARs are modular synthetic receptors consisting of an antigen-binding domain that confers antigen-specific binding, an extracellular hinge region that extends the binding units from the transmembrane region, a transmembrane domain anchoring CAR to the T-cell membrane, and intracellular signalling domains [15]. 

To date, six CAR T (CART) cell therapies have been approved by the FDA [16,17]. Tisagenlecleucel (target antigen CD19) was used for the treatment of B-cell acute lymphoblastic leukemia (ALL) with an overall survival rate of 84%; event-free survival was 69% among children and young adults with relapsed or refractory B-cell ALL [18]. Axicabtagene ciloleucel (target antigen CD19) addresses B-cell non-Hodgkin lymphoma (NHL) and follicular lymphoma and is a second-line therapy for large B-cell lymphoma, with an estimated overall 2-year of 61% [19]. Brexucaptagene autoleucel (target antigen CD19) treats mantle cell lymphoma and B-cell non-Hodgkin lymphoma [20]. At a median follow-up of 3 months, the objective response rate in mantle cell lymphoma was 94% for blastoid or pleomorphic variants and 82% for *TP53*-altered variants [20]. Lisocabtagene maraleucel (target antigen CD19) was used for the treatment of B-cell non-Hodgkin lymphoma. In adults with refractory or relapsed B-cell non-Hodgkin lymphoma, the median event-free survival was 95% (6 months follow-up) [21]. Idecabtagene vicleucel (target antigen B cell maturation antigen) was used to treat relapsed or refractory multiple myeloma, with 33% complete response among the treated patients [22]. Ciltacabtagene autoleucel (target antigen B cell maturation antigen) was used for the treatment of relapsed or refractory multiple myeloma, with 67% of the patients achieving stringent complete response [23].

## 3. Molecular Biological Aspect of T-Cell Therapy

It is well known that the lymphopenic state facilitates graft acceptance by providing a niche for TILs to expand, although the mechanism was elusive until the 1980s [1]. Endogenous cells from an immunoreplete host can hinder ATC via three mechanisms. Immune cells such as T cells, B cells, and NK cells compete for cytokines such as IL-2, IL-7, and IL-15 that help in immune activation. CD4^+^CD25^+^FOXP3^+^ T regulatory (T_reg_) cells present in immunocompetent hosts confer immunosuppression by direct cell-to-cell contact or by releasing immunosuppressive cytokines. Immature antigen-presenting cells might confer anergy to transferred T cells, reducing the antitumour activity. Additionally, the tumour itself plays an active role in immune evasion by releasing molecules that avoid immune cell attack and by presenting low MHC recognition sites, minimizing competitive tumour recognition. Clinically, it has been found that lymphodepletion results in significant diminution of inhibitory elements [1]. Thus, following the isolation of tumour-infiltrating lymphocytes from biopsy samples and subsequent ex vivo expansion using IL-2, TILs are infused into a patient who has undergone lymphodepletion.

Despite successful transfusion of T cells, binding to MHC was still insufficient to mediate signalling. To overcome this limitation, the players in T-cell activation were identified as the TCR heterodimer-engaging antigen and the cluster of differentiation 3 (CD3). The CD3 complex is composed of a homodimer of CD3ζ and heterodimers of CD3ε, CD3δ, CD3ε, and CD3γ [24]. Tyrosine-based activation motifs present in CD3 polypeptides initiate the activation of T cells. Initially, biologists cloned chimeric CD3ζ chains with antibody crosslinking [24]. Later, a modified TCR-like molecule was created by fusing ζ-chain receptors to a single-chain variable fragment (scFv) acting as its extracellular domain. CARs utilize scFv or alternative ligands to bind target antigens [24]. Thus, CARs are independent of binding to MHC, unlike physiological TCR. This genetic engineering approach provides several advantages. First, bulk T-cell genetic modification with CAR rapidly provides ample antigen-targeted cells in a short time. Second, CAR (HLA-independent)-engineered cells work without tumour MHC expression status. Third, synthesising CAR is facilitated by the flexibility of antibody generation by heavy- and light-chain library screening. Finally, CARs are modular, synthetic protein molecules that utilize CD28; CD19; 4-IBB costimulatory domains; and stimulatory domains such as OX40, CD27, ICOS, and NKG2D, which, interestingly, lead to the production of canonical receptors CD28/CD3ζ and 4-IBB/CD3ζ [24,25,26,27,28].

CART cell production starts with the T cells collected from the patients after undergoing leukapheresis. T cells are first collected; then, CD3 and or CD28 activation is performed, resulting in T-cell activation and proliferation. Thus, the susceptibility towards transduction is enhanced. The expansion of the transduced (retroviral transduction system using γ retroviral or lentiviral system) cells continues until the achievement of the expected dosage of CART cells. The generated CART cells undergo QC/QA quality control and validation of their function and sterility prior to clinical use [29].

## 4. Role of T-Cell Therapy in Head and Neck Cancer

The FDI has approved CART cell therapy for the treatment of haematological neoplasms [30]. CAR-MUC1-IL22T showed significant cytotoxicity in head and neck squamous cell carcinoma (HNSCC) [31]. Based on cancer genome atlas analysis of the characteristics of HNSCC, CD70 was selected to determine the efficiency of CART cell therapy in in vitro assays [32]. The results suggested the CART cell therapy targeting CD70-positive HNSCC accomplished the objective, sparing the CD70-negative HNSCC cells [32]. Therefore, CD70/CART cell therapy is possibly not appropriate for generalized treatment of HNSCC but promising for future clinical trials for CD70-positive HNSCC cases. Approximately 10–15% of HNSCC cases reported NOTCH 1 mutations, making them suitable for CART cell therapy [32]. Implantation of synNOTCH into CART cells enables recognition of target antigens on the surface of the solid tumour cells. Additionally, it locates and kills adjacent cancer cells. The injection of synEGFRvIII/CART in glioblastoma mouse models resulted in higher tumour recognition ability and T-cell durability compared to conventional CART cells. Prostate-specific membrane antigen and overexpression of dominant negative TGFβ receptor II (TGFβRII) blockade result in higher antitumour efficiency. The feasibility and safety of such a combination was assessed by phase I clinical trials [32].

ATC transfer treatment for head and neck cancer is broadly classified into human papilloma virus (HPV)-associated HNSCC and non-HPV-associated HNSCC. HPV-associated oropharyngeal cancer harbours viral antigens that are ideal targets for the ATC transfer protocol. E6 and E7 viral proteins are targeted by T cells. Autologous TIL generation was performed from T cell subcultures, which are reactive towards E6/E7 protein. Initial results showed complete tumour regression following TIL therapy led to the multicentre, multicohort, non-randomised clinical trial to test TIL (LN-145) in recurrent and metastatic head and neck metastatic squamous cell carcinoma (NCT03083873) [30].

Non-invasive, high-precision procedures involving genetically engineered T cells are the latest development in head and neck cancer treatment. HPV16E6 peptide (HLA-A*02:01) was identified among tumour-infiltrating lymphocytes in a patient with metastatic HPV-associated cancer, leading to the first human clinical trial using engineered T-cell transfusion to treat HNSCC [33]. All patients demonstrated high peripheral blood engraftment of E6 TCR-T cells. Objective tumour response was observed in 17% patients, with none of the patients showing autoimmune, acute, or dosage-related toxicities. Furthermore, improved outcome was noted during the E7 TCR-T phase I clinical trial, with 50% of patients showing objective tumour response [33]. This is possibly because the E7 epitope that is targeted by this TCR is highly conserved across all the strains of HPV16.

ATC for non-HPV-associated HNSCC can be broadly divided into two approaches: Epstein Barr virus (EBV) T cells and cancer germline antigens. Cancer germ lines lack MHC I expression, which makes them rational targets for TCR T-cell therapy. Previously, cancer a germ line T-cell approach has been successful in melanoma and synovial cell sarcoma. Melanoma-associated antigen 4 and Kyushu lung cancer antigen 1 are the target antigens for future head and neck cancer germline antigen therapy [34].

## 5. Limitations of Adoptive T-Cell Transfer

The greatest limitation of ATC is the development of tachyphylaxis for single-antigen-targeting CAR constructs. The tachyphylaxis phenomenon is known as antigen escape in the world of immunology [35]. CD19/CART therapy showed optimum tumour response initially when relapsed or refractory patients were treated. However, subsequent treatment with the same combination resulted in downregulation of CD19 antigen in nearly two-thirds of the patients. Glioblastoma, an example of head and neck cancer, showed similar IL13/CART cell antigen escape [36]. This led to further optimisation of the ACT protocol and the introduction of dual CAR constructs or tandem CARs consisting of scFvs for concomitant targeting of multiple tumour antigens [36]. Both strategies proved to be better than the single antigen-targeting protocol. CD19/CD22, CD19/BCMA, and HER2/IL13Ra2 have been used for the treatment of diffuse B-cell lymphoma, multiple myeloma, and glioblastoma, respectively. Another challenge of ATC is the offset effect, particularly in solid tumours [37]. Solid tumour antigen expression is also present in its healthy counterpart. Tumour-restricted post-translational modifications such as Tn (GalNAca1-o-Ser/Thr) and sialyl-Tn (STn) (NeuAca2-6-GalNAca1-O-Ser/Thr) are among the strategies for overcoming this obstacle [38]. CART cell trafficking is another reason for lower efficacy in solid tumours. The penetration of CART cells into the tumour is diminished due to the presence of immunosuppressive tumour microenvironment tumour stroma (physical barrier). Alternate delivery routes mitigate these limitations. Engineered CART cells expressing heparinase and integrin αvβ6-CART cells modified to express CXCR2 enhance the penetration and trafficking of CART cells [39].

Unfortunately, CART-cell-related toxicities have never been used as a first-line treatment option for cancer, despite excellent performance during clinical trials that potentially revolutionized the cancer treatment strategies. In acute lymphoblastic leukemia (ALL), nearly half the patients undergoing FDA-approved CD19/CART cell therapy suffered from cytokine release syndrome, which is associated with hyperproduction of cytokines and massive T-cell expansion (hemophagocytic lymphohistiocytosis and macrophage activation syndrome), potentially leading to life-threatening capillary leak with hypoxia and hypotension [40].

Overall, the immunosuppressive tumour microenvironment poses a major impediment to the success of adoptive T-cell transfer. Myeloid-derived suppressor cells and regulatory T cells are examples of cell types that drive immunosuppressive action in the tumour microenvironment. PD1 or CTLA4 serve to decrease antitumour immunity [41].

## 6. Molecular Biological Mechanisms of CTLA4 and PD1/PD-L1

**Figure 1:**
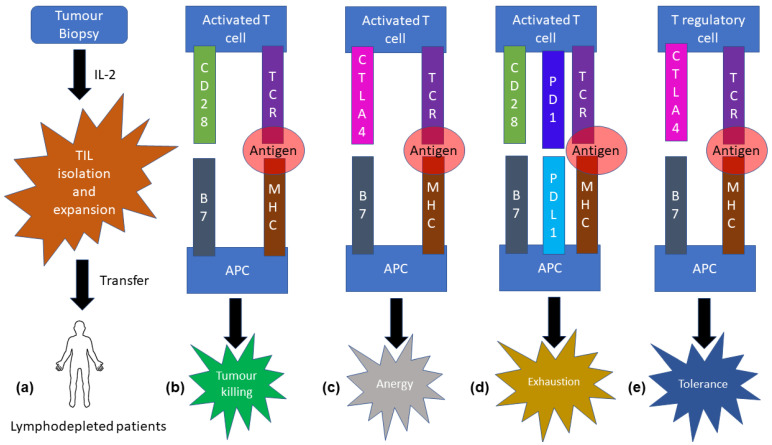
Simplified schematic representation of molecular biological aspect of immunotherapy. (**a**) Mechanistic approach of adoptive T-cell transfer. Tumour-infiltrated lymphocytes are isolated and expanded with the help of IL-2, then transfused to lymphodepleted patients. (**b**) Antigens bound to MHC present in APC interact with TCR, and CD28 binds with the B7 receptor, leading to the release of perforin and granzyme (not shown in the figure), resulting in tumour death. (**c**) CTLA4 competitively binds with the B7 receptor in APC, causing T-cell anergy. (**d**) PD1 and PD-L1 binding cause T-cell exhaustion. (**e**) T regulatory cells also harbour CTLA4, which binds with B7 receptors of APC, causing T-cell tolerance. Thus, the inhibition of checkpoint receptors can reverse immunosuppression.

CTLA4 is a new member of the immunoglobulin superfamily, with structural and biochemical similarities to CD28. CTLA4 is found in chromosome 2 (2q33.2) and is selectively expressed in immune cells [42]. Strong induction of CTLA4 occurs after antigenic stimulation. T_reg_ cells that have an immunosuppressive function express high levels of CTLA4. CTLA4 forms membrane-bound homodimers consisting of an extracellular immunoglobulin-like domain, a transmembrane region, and an intracellular cytoplasmic tail that can recruit signalling molecules and orchestrate surface expression [42]. Lipopolysaccharide-responsive and beige-like anchor protein (LRBA) controls the trafficking of CTLA4 (post-activation)-containing vesicles on the cell surface [43]. CTLA4 binds to B7-1 (CD80) and B7-2 (CD86) ligands that are expressed by dendritic cells (Figure 1).

Both CTLA4 and CD28 bind to B7 ligands, although CTLA4 has greater affinity towards B7 compared to CD28 [1]. CD28 and CTLA4 have opposite immunoregulatory functions (Figure 1a–c). T-cell activation and proliferation is inhibited by CTLA4. Similar observations were made during in vivo experiments on *Ctla4*-knockout mice showing signs of T-cell-mediated lymphoproliferative autoimmune disorder. Conversely, amelioration of the disorder was observed when CTLA4:Fc fusion protein was introduced in the same disease models. This entire process of lymphoproliferative disorder was seen to be controlled by CD28. Moreover, exhibition of similar severe multiorgan lymphoproliferative infiltration occurs in CTLA4-deficient patients. FDA-approved abatacept (CTLA4Ig) is used to treat these patients [44].

CTLA4 downregulates T-cell activation by antagonising CD28 at the immunological synapse (interface between lymphocytes and antigen-presenting cells that controls antigen-induced signalling) by costimulatory ligand competition. CTLA4 mediates the internalization of ligands, thus inhibiting CD28-B7 binding. CTLA4 can reorganise the cytoskeleton and perturbs antigen-presenting cell–T cell conjugation. Finally, CTLA4 inhibits T-cell activation and proliferation by recruitment of inhibitory effector molecules such as SH2 domain-containing tyrosine phosphatase 2 (SHP2), which inhibits the phosphorylation of the CD3ζ subunit [45].

**Figure 2:**
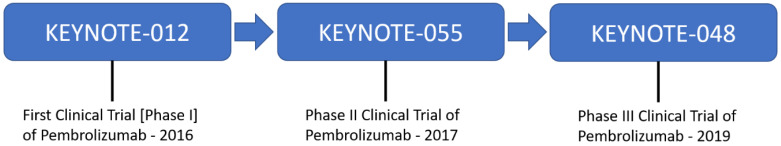
Schematic representation of the pembrolizumab clinical trials, which started in 2013 and ended in 2019, with phase I ending in 2016, phase II ending in 2017, and phase III ending in 2019. The successful outcome of these clinical trials resulted in FDA approval of pembrolizumab for the treatment of HNSCC.

Initially, PD1/PD-L1 was identified as a putative mediator of apoptosis [46]. Now, it is considered analogous to CTLA4. It binds to B7-H1 (B7 homologous PDL1) and B7-DC (B7 homologous PDL2). Phosphorylation of immunoreceptor tyrosine-based switch motif and inhibitory motif of PD1 results in the recruitment of SHP1 and SHP2, which dephosphorylate and inactivate the CD3ζ subunit of TCR, ultimately resulting in inactivation of T cells [47,48] (Figure 2d). Additionally, it has been found that both CTLA4 and PD1 ultimately impact glucose uptake and utilization by inhibiting protein kinase B [49].

The molecular biological pathways elucidating the precise mechanism by which PD1 mediates apoptosis or T-cell exhaustion is still elusive. Hypothetically, phosphoinositide 3 kinase signalling interacts with mitochondrial B-cell lymphoma extra large (Bcl-xL), acting as a critical control point where PD1-mediated PI3K inhibition downregulates Bcl-xL and ultimately resulting in apoptosis. PDL1 on dendritic cells can also control T-regulatory cell differentiation and lead to immunosuppressive activity. Neoplastic cells can exploit this mechanism, inducing T cell exhaustion (Figure 1d) and reduced antitumour immunity by upregulating PD1 ligands [49].

## 7. Immune Checkpoint Therapy

The FDA approved has a number of immune checkpoint inhibitors for cancer treatment, as described in Table 1.

Ipilimumab (monoclonal antibody) is a CTLA4-blocking antibody approved by the FDA for the treatment of late-stage melanoma based on the excellent outcome of the MDX010-020 trial [50]. Of the 676 patients treated with ipilimumab, 474 patients were available to study the tumour responses and survived beyond 2 years from the date of treatment. One-fourth of these 474 patients survived ≥2 years after treatment with ipilimumab alone, 19% survived ≥2 years when treated with a combination of ipilimumab and the gp100 vaccine, and 17% survived ≥2 years when treated with the gp100 vaccine alone. Among the survivors (≥2 years), nearly half of the survived ≥3 years [50]. FDA further approved ipilimumab for the treatment of metastatic melanoma in adults and paediatric patients. The FDA also approved combination therapies including ipilimumab and nivolumab for BRAF V600 unresectable or metastatic melanoma and renal cell carcinoma [51].

Nivolumab (monoclonal antibody against PD1) was approved for the treatment of metastatic melanoma patients who were previously treated with ipilimumab. The FDA subsequently approved nivolumab and ipilimumab combination therapy for the treatment of metastatic melanoma (positive lymph node) or complete resection melanoma patients [52]. Improved progression-free survival resulted in the FDA approving the treatment of recurrent or metastatic melanoma as a first-line treatment, irrespective of BRAF V600 mutation status. Additionally, nivolumab obtained approval for the treatment of squamous non-small cell lung cancer, non-squamous non-small cell lung cancer, small cell lung cancer, advanced renal cell cancer, Hodgkin’s lymphoma (relapsed post-autologous stem cell therapy), relapsed/refractory metastatic HNSCC, unresectable metastatic urothelial cancer, metastatic colorectal cancer (microsatellite instability-high or mismatch repair-deficient), and hepatocellular carcinoma [53,54,55,56,57].

Another PD1 inhibitor is pembrolizumab. It was first approved for the treatment of metastatic melanoma that is refractory to CTLA4 and BRAF inhibitors. Patients receiving pembrolizumab showed an overall response rate of 24% (NCT01295827). The treatment was well tolerated, with no death and most common drug-related untoward effects being pruritis, rash, and fatigue [58]. 

The FDA approved yet another PD-1 inhibitor, cemiplimab, for the treatment of locally advanced metastatic cutaneous squamous cell carcinoma. The overall response rate in a phase I clinical trial was 50%, which is similar to the response in the phase II clinical trial (metastatic disease cohort). Untoward effects such as nausea, diarrhea, and fatigue occurred in 15% of patients, forcing 7% patients to completely discontinue treatment [59].

Besides PD1 blockers, the FDA has also approved a number of PD-L1 blockers. Avelumab was the first drug approved for the treatment of Merkel cell carcinoma. Approximately 32% of patients showed an overall response, with few events of adverse drug reactions observed, including lymphopenia, enterocolitis, infusion-related colitis, synovitis, and interstitial nephritis [60].

The FDA approved durvalumab for the treatment of urothelial bladder cancer. The overall response rate was 31% in response evaluable patients, with nearly half of patients reported to be histologically PD-L1-positive and no response recorded in PD-L1-negative patients [61]. Subsequently, atezolizumab was also approved for the treatment of metastatic urothelial cancer [62].

## 8. Role of Immune Checkpoint Therapy in Head and Neck Cancer

Pembrolizumab, a PD-1 inhibitor, was the first drug to be approved by the FDA (Figure 2) in 2016 following several clinical trials.

An open-label, multicentre, phase 1b clinical trial was conducted with patients with HNSCC. All patients were adults and preferably histologically PD-L1-positive. Patients were intravenously treated with 10 mg/kg body weight pembrolizumab at an interval of every 2 weeks. Response evaluation criteria in solid tumours (RECIST) were used to assess safety in each treated patient. Overall, pembrolizumab was well tolerated among the treated patients. Approximately 20% of patients showed signs of grade 3–4 drug-related adverse reactions, with increased levels of alanine aminotransferase and aspartate aminotransferase, as well as hyponatremia. An overall response was observed in approximately one-fifth of the patients. Pembrolizumab also proved to be effective in HPV-positive patients, with 25% of patients showing an impressive response [63].

KEYNOTE-055, a single-arm phase II study, was conducted to assess the benefit of pembrolizumab at a dose of 200 mg IV every 3 weeks on HNSCC patients who are refractory to platinum-based therapy and cetuximab (Figure 1). The overall response rate was only 16%, and the duration of response was 8 months [64].

The KEYNOTE-048 phase III clinical trial of pembrolizumab in recurrent or metastatic HNSCC was conducted to analyse the efficacy of the drug by assessing the PD-L1 combined positive score (CPS) (Figure 1). Approximately 14% patients had PDL-L1 CPS < 1, and 42% had PDL1 CPS 1-19. The clinical trial clearly exhibited a better overall response rate and disease-free survival compared to the cetuximab chemotherapy subgroup. The median overall survival was improved under pembrolizumab chemotherapy versus cetuximab chemotherapy. The pembrolizumab chemotherapy treatment group showed improved survival relative to the PD-L1 CPS 1-19 subgroup [65]. However, HNSCC treatment with tavokinogene telseplasmid with electroporation, pembrolizumab, and epacadostat (inhibitor of indoleamine 2.3 dioxygenase) was terminated due to ineffective outcomes (NCT03823131), and the same treatment for melanoma (NCT04526730) has an estimated study completion date of November 2023.

Furthermore, a phase I trial of cetuximab (an anti-EGFR monoclonal antibody), radiotherapy, and ipilimumab (anti anti-CTLA4 monoclonal antibody) was completed to treat locally advanced head and neck cancer from 2013 to 2016. The preliminary results were very promising, with nearly 75% patients showing disease-free survival for three years. However, the expression of PD1/LAG3/CD39 negatively impacted the disease-free survival of the treated patients [66], so cetuximab and ipilimumab are not FDA-approved for the treatment of HNSCC.

Nivolumab is another FDA-approved drug for the treatment of HNSCC in patients who are refractory to platinum-based chemotherapy. CheckMate 141, a randomised, open-label, phase 3 trial was conducted to assess the efficacy of nivolumab. Patients received nivolumab at a dose of 3 mg/kg body weight at an interval of 2 weeks versus standard chemotherapy (methotrexate, docetaxel, or cetuximab). Patients who received standard chemotherapy survived 2 months fewer than patients who received nivolumab. The overall response rate of nivolumab was 13.3%, compared to 5.8% in the standard therapy group. Nearly one-third of patients treated with nivolumab suffered from drug-related adverse effects compared to standard therapy groups [54].

## 9. Limitations of Immune Checkpoint Therapy

Durability of overall tumour response is a hallmark of immunotherapy that can be translated into prolonged survival. FDA-approved immune checkpoint inhibitors raise the tail of patient survival curves. However, few patients are responsive to this treatment, which can be attributed to various factors.

Tumours are heterogeneous in nature, consisting of various immunosuppressive cells and inhibitory cytokines that undermine antitumour immune response. T regulatory infiltration into the tumour microenvironment confers an immunosuppressive effect, as the immune checkpoint blockade effect largely depends on the ratio of T effector and T regulatory cells [67,68] (Figure 1e).

Tumour immunogenicity is another result of high tumour mutational burden, leading to intratumour heterogeneity. This ultimately impacts the selection of immunogenic subclones. Additionally, genetic instability due to DNA repair alteration and replication genes leads to the formation of subsequent neoantigens. Paradoxically, the expression of interferon γ increases the expression of PD-L1, resulting in adaptive resistance of tumour cells [67,68].

Few melanoma cases demonstrate impaired priming of naïve T cells via suppressed antigen-presenting cell recruitment, resulting in fewer tumour-infiltrating lymphocytes and resistance to immune checkpoint blockade. Abrogation of β2-microglobulin function impacts proper folding of MHC I, assisting tumour cells in going unnoticed by immune surveillance. Additionally, coexpression of alternate immune checkpoint receptors such as B and T lymphocyte attenuator (BTLA), T-cell immunoreceptor tyrosine-based inhibition motif domain (TIGIT), and V-domain immunoglobulin-containing suppressor of T-cell activation (VISTA) confer T-cell exhaustion [67].

Loss of tumour suppressor phosphatase and tensin homolog (PTEN) results in a reduction in tumour-infiltrating lymphocytes in the tumour microenvironment via vascular endothelial growth factor expression. Presumably, PI3Kγ increases myeloid-derived suppressor cells (MDSCs) in the tumour microenvironment. As β-catenin is negatively correlated with TILs, higher expression of β-catenin results in impaired priming of naïve T cells [68].

The involvement of chromatin remodelling in immune checkpoint blockade has emerged recently. BRG1- polybromo-, and BRG1-associated factors are chromatin remodellers and tumour suppressors. Mutation of such chromatin remodellers resulted in increased sensitivity to PD-L1 and CTLA4 blockade [67].

Ipilimumab-related adverse effects occurred in more than 70% patients. Enterocolitis, hepatitis, and dermatitis were commonly observed. Nearly 50% patients suffered from skin rashes. Life-threatening hepatotoxicity occurred in 10% of patients. Some of the patients suffered from hypophysitis with adrenal insufficiency. Few rare complications, such as Guillan–Barré syndrome, severe motor neuropathy, myasthenia gravis, aseptic meningitis, and optic neuritis, were reported. Pneumonitis was rarely reported in cases of pembrolizumab treatment, although 10% of patients receiving PD1/PD-L1 suffered from pneumonitis. PD1/PD-L1 showed a different toxicity profile compared to CTLA4 inhibition treatment. Critical neurological and endocrinological untoward effects were observed in children receiving PD-L1 treatment [69].

## 10. Interleukin Biology

Inflammation by immune cells designed to restrict the spread of infection and primary healing of wounds can result in their unpremeditated assistance of multiple cancer hallmarks, consequently exacerbating inflammatory elements and promoting cancer progression. IL-1 has been associated with cancer progression. IL-1α and IL-1β, also known as alarmins, initiate and amplify inflammation [70].

Pathogen-associated molecular patterns (PAMPs) lead to persistent inflammation, and damage-associated molecular patterns (DAMPs) trigger nuclear factor kappa B (NF-κB), which primes pro-IL-1β and nucleotide-binding oligomerisation domain-like receptor (NLR). The end result is the release of IL-1β from fibroblasts, epithelial cells, and antigen-presenting cells [71]. IL-1β induces the production of reactive oxygen species (ROS), which causes DNA damage and promotes the synthesis of IL-6 and IL-11 from epithelial and myeloid cells and IL-22 from lymphoid cells and γδ T cells. In neoplastically transformed cells, IL-6, IL-11, and IL-22 phosphorylate and activate signal transducer and activator of transcription factor 3 (STAT3), which plays an anchor role in proliferation, restriction of apoptosis, epithelial–mesenchymal transition, and metastasis [72].

Recent developments in cancer research highlight the importance of immune evasion driving metastasis. Metastasis largely depends on cancer cell-intrinsic and -extrinsic cytokine signalling. Overexpression of cytokines such as IL-6 and IL-11 activates the PI3K-AKT-mTOR pathway, resulting in upregulation of glycolysis; metabolic reprogramming; and upregulation of NF-κB, rat sarcoma (RAS), rapidly accelerated fibrosarcoma (RAF), mitogen-activated protein kinase (MAPK), and STAT3. These pathways drive proliferation and reduce apoptosis, epithelial–mesenchymal transition (EMT) and metastasis, along with overproduction of IL-8, metalloproteinases and vascular endothelial growth factor, leading to angiogenesis [70]. EMT is also induced by cytokines such as IL-1β, IL-13, IL-17, IL-22, IL-23, and IL-35. Additionally, the recruitment of polymorphonuclear leukocytes is facilitated by IL-8. Together with monocytes, they differentiate into myeloid-derived suppressor cells (MDSCs) that inhibit T_H_1 response. MDSCs lead to tumour-associated macrophages (TAMs), which contribute to the pool of transforming growth factor β (TGFβ), resulting in an immunosuppressive tumour microenvironment. Subsequently, TGFβ, together with IL-33, induces differentiation of T_reg_, which harbours high-affinity IL-2 receptors, representing a major source of IL-10, which suppresses antitumour immune response. Furthermore, TGFβ, together with IL-6, promotes expansion of T_H_17, which upregulates IL-17, further promoting recruitment of MDSCs [70]. A comprehensive summary of how different interleukins affect cancer has been collated (Table 2).

Interleukins not only take part in the progression of cancer but also restrict it. NK cells recognise and eliminate cancer cells because of the presence of specific receptors. DAMPs that are released from neoplastic cells are processed by antigen-presenting cells that, in turn, synthesise IL-12 and IL-15 [70]. These interleukins trigger the cytotoxic activities of NK and cytotoxic T lymphocytes (releasing granzymes and perforins to kill tumour cells) and induce the release of IFNγ (inducing apoptosis of tumour cells). Dendritic cells migrate to lymph nodes and present MHC II receptors to the naïve T cells. Thus, they initiate activation of lymphocytes. It should be noted that the naïve T cells originate from lymphoid progenitors with the help of IL-3/IL-7. The dendritic cells also release IL-12, which triggers T-box transcription factor T-bet, leading to T_H_1 polarization. T_H_1, together with naïve T cells, upregulates IL-2 in tumours, resulting in rapid proliferation of lymphocytes, thereby killing the tumour cells [70] (Table 2).

## 11. Interleukin Therapy

To date, no interleukin has been approved as a biomarker by the FDA despite reports suggesting several interleukins as biomarkers. Several preclinical and clinical studies were conducted to determine the progression of cancer using interleukins. IL-1β neutralization was achieved through the canakinumab anti-inflammatory thrombosis outcome study (CANTOS) [109]. Canakinumab-treated patients showed significant reduction in overall cancer mortality. A canakinumab phase III clinical trial to treat non-small cell lung cancer (NCT03447769), a phase II clinical trial to treat myeloblastic syndrome and chronic myelogenous leukemia (NCT04239157), phase III clinical trial in combination with pembrolizumab and chemotherapy in non-small cell lung cancer (NCT03631199), and a phase III clinical trial in combination with standard chemotherapy for the treatment of non-small cell lung cancer (NCT03626545) are ongoing. 

IL-1R antagonist anakinra, which is FDA-approved for the treatment of rheumatoid arthritis and neonatal-onset multisystem inflammatory disease, has also been under trial for multiple cancers, and outcomes are awaited.

Siltuximab (anti-IL-6 antibody) showed promising preclinical results in the xenograft cholangiocarcinoma model [110] but did not achieve an impressive outcome in multiple myeloma clinical trials in combination with bortezomib [111]. Theoretically, neutralization of IL-6 might help immune checkpoint inhibitor therapy. In contrast, it has been observed that IL-6 inhibitors downregulate PD1/PDL-1 and might decrease the effectiveness of immune checkpoint inhibitors [112]. Further studies are warranted to evaluate the effectiveness of this antibody.

HuMax-IL-8 (IL-8 inhibitor) has not shown much promise as a monotherapy. Therefore, clinical trials combination with nivolumab (NCT03400332, NCT04123379, and NCT040\50462), nivolumab and degarelix (gonadotropin-releasing hormone antagonist) (NCT03689699), and nivolumab and stereotactic radiotherapy (NCT04572451) are underway.

Preclinical models of gastric cancer and prostate cancer showed significant reduction in tumour burden after IL-17 neutralizing therapy in combination with anti-PD-1 [113]. Another study showed a reduction in multiple myeloma after IL-17 neutralization [114], which triggered the initiation of a clinical trial (NCT03111992) to determine the efficacy of IL-17 neutralization using antibody CJM112. 

IL-23 (derived from MDSCs) activates the androgen receptor pathway, supporting proliferation and avoiding apoptosis in androgen-deprived conditions such as castration-resistant prostate cancer. A preclinical model of prostate cancer showed promising outcome after neutralization of IL-23 [115]. A phase I/phase II clinical trial (NCT04458311) is estimated to be complete in October 2024 using tildrakizumab (anti-IL-23 antibody) in combination with abiraterone acetate (androgen synthesis blocker) for the treatment of prostate cancer. 

IL-2 rapidly proliferates lymphocytes that kill tumour cells, although at low abundance. IL-2 preferentially binds to T_reg_ cells that aid in immunosuppression. This has led to the development of several engineered IL-2 clones with amplified affinity towards the CTL-expressed IL-2Rβ-γc complex [70]. Bempegaldesleukin (non-α IL-2 variant) in combination with immune checkpoint inhibitors is in a phase III clinical trial for the treatment of bladder cancer (NCT04209114), renal cell cancer (NCT03729245), and melanoma (NCT03635983). A phase II clinical trial was completed in February 2022 (NCT04144517) using ALKS4230 (non-α IL-2 variant) in combination with pembrolizumab for the treatment of non-cutaneous head and neck squamous cell carcinoma; however, results have yet to be released.

Other key interleukins undergoing clinical trials are IL-10, IL-12, IL-15, IL-23, and IL-36 [71]. In a phase I clinical trial, pegilodecakin (PEGylated IL-10 variant) with immune checkpoint inhibitors proved successful, although follow-up clinical trials (NCT03382912 and NCT03382899 in combination with nivolumab and pembrolizumab, respectively, were stopped due to an unfavourable risk-versus-benefit ratio. IL-12 treatment in combination with pembrolizumab yielded a complete response rate of 36%. A phase II clinical trial in triple-negative breast cancer (NCT03567720) has an estimated study completion date of August 2024. ALT-803 (IL-15 superagonist) in combination with bacillus Calmette-Guérin (BCG) treatment resulted in complete response in bladder cancer [87] and has now progressed to a phase II clinical trial (NCT02138734) (estimated completion in December 2027). Finally, IL-36γ in combination with IL-23 and OX40 ligand is currently undergoing a phase I open-label multicentre dose escalation clinical trial (NCT03739931) followed by dose expansion in triple-negative breast cancer, HNSCC, urothelial cancer, immune checkpoint refractory melanoma, and non-small cell lung cancer lymphoma (expected completion in April 2025) [70].

## 12. Limitations of Interleukin Therapy

The major limitations of interleukin therapy are high toxicity, poor therapeutic index, ability to induce immunosuppressive response via T_reg_ cell expansion, and short circulating half-life [70,73,74,75,76,77,78,79,80,81,82,83,84,85,86,87,88,89,90,91,92,93,94,95,96,97,98,99,100,101,102,103,104,105,106,107,108]. Dose-limiting toxicities resulted in hypotension, vascular leak syndrome, oedema, cardiac arrythmias, haematological, and renal toxicities. Lowering the dose resulted in less toxicity but reduced efficacy compared to the high-dose intravenous regimen. To mitigate dose-limiting toxicities, some approaches adopted by various researchers include (a) altering interleukins to transform less toxic molecules, allowing higher doses, and (b) altering the administration route. Altering the route of administration significantly impacts toxicity. Intravenous bolus dosing results in peak serum concentration, albeit with rapid clearance, whereas subcutaneous administration results in low serum concentration but sustained exposure. 

Intralesional administration of interleukins for the treatment of melanoma proved promising, with lower toxicity and complete response in approximately two-thirds of the patients. The intralesional approach demonstrated superior efficacy relative to all other routes of administration. However, this approach itself is subject to the inherent limitation of a cumbersome dosing regimen necessary in metastatic lesions, as the effect of the treatment is confined to the treated sites and does not appear to improve untreated sites. Combination therapy approaches such as the combination of interleukins with IFN α or conventional chemotherapeutic agents have not provided a conclusive beneficial overall outcome compared to the conventional interleukin systemic administration approach. 

Interleukins confer both immunostimulatory and immunosuppressive action. At high doses, interleukins act as immune activators via expansion of CD4^+^, CD8^+^, and NK cells, whereas at low doses, they become immunosuppressive by triggering the expansion of T_reg_ cells [70]. Therefore, ongoing strategies to develop modified forms of interleukins that function in a single capacity (immunosuppressive or immune activator) for the treatment of autoimmune disorders and cancers can help to improve interleukin efficacy. 

Rapid clearance of high-dose interleukins via the kidney results in a short half-life and further limits interleukin treatment. Optimisation of pharmacokinetic properties of protein therapeutics can result in improved efficacy. Extension of half-life can be accomplished by modifying the overall charge of the protein molecule, increasing the hydrodynamic radius/molecular weight and fusion to protein domains.

## 13. Cancer Vaccines

Cancer vaccines trigger the immune system to protect against the development of cancer and can be broadly classified into two types: prophylactic and therapeutic [1]. Vaccines against hepatitis B and human papilloma virus reduced the incidences of hepatocellular carcinoma and HPV-associated cervical carcinoma [1]. In contrast, therapeutic vaccines harness the immune system to eliminate neoplastically transformed cells. Bacillus Calmette–Guérin (BCG) vaccine is used to treat bladder cancer [87]. To date, four vaccines have been approved by the FDA to prevent cancers. Cervarix (a bivalent vaccine) was approved for the prevention of cervical cancer caused by HPV types 16 and 18 and can therefore help prevent HPV-related anal, cervical, head and neck, penile, and vulvar and vaginal cancer. Gardasil (quadrivalent vaccine) protects against HPV 16, 18, 6, and 11 and prevents HPV-related anal, cervical, head and neck, penile, vulvar, and vaginal cancer. Gardasil-9 (nine-valent vaccine) protects against HPV 16, 18, 31, 33, 45, 52, and 58 and prevents HPV-related anal, cervical, head and neck, throat, penile, vulvar, and vaginal cancer [116]. A prophylactic effect, especially for oropharyngeal cancer, is based on clinical evidence of prevention of oral HPV infection and HPV-related cellular changes that include benign and precancerous lesions and conditions. The hepatitis B vaccine (HEPLISAV-B) protects against hepatitis B viral infection and prevents hepatitis B virus-related hepatocellular carcinoma. To date, two therapeutic cancer vaccines have been approved by the FDA: Bacillus Calmette–Guérin (BCG) vaccine for the treatment of early stage bladder cancer and Sipuleucel-T (Provenge) (composed of patients stimulated antigen presenting cells) for the treatment of prostate cancer [1].

## 14. Role of Cancer Vaccines in Head and Neck Cancer

HPV-16 is associated with 90% of HPV-related oropharyngeal cancer cases. Other HPV serotypes such as 31, 33, 35, 39, 45, 51, 52, 56, 58, 59, 68, 73, and 82 have been recognised as oncogenic [117]. Expression of E6 and E7 proteins by HPV cause degradation of p53 and the retinoblastoma gene, resulting in uncontrolled cellular proliferation [117]. HPV prophylactic vaccines are mainly composed of viral particles containing L1 proteins that induce the production of IgG antibodies against HPV. However, these vaccines are inefficient following HPV infection because L1 proteins are no longer expressed in the oncogenic state of HPV. HPV vaccines are highly efficient in preventing more than 90% of HPV-induced head and neck precancer lesions [117].

Oncoproteins E6 and E7 are ideal targets for the development of therapeutic vaccines for HPV-positive HNSCC. This strategy has been tested in anogenital cancer. Approximately 79% patients with HPV-16-positive vulvar grade III intraepithelial neoplasia (among them, nearly half of the patients showed complete response) responded to the vaccine [118]. Some notable clinical trials using MEDI0457 (a DNA-based vaccine against E6/E7-HPV-16/18 encoding IL-12) in combination with durvalumab (NCT03162224) showed initially positive outcomes. Additionally, promising clinical trials including NCT02002182 and NCT04287868 open new possibilities for future translational research.

Apart from HPV, EBV induces undifferentiated nasopharyngeal carcinoma. Live modified vaccinia Ankara virus was used in a phase I clinical trial (NCT0114799), revealing that the vaccine has few adverse effects [117]. A phase II clinical trial testing the efficacy of the vaccine is currently underway (NCT01094405).

Phase I and II clinical trials are currently underway for the following therapeutic vaccines targeting non-viral antigens in HNSCC: p53 (NCT00404339), suvivin-2B (UMIN000000976), HLA-B7/β2 (NCT00050388), LY6K, Telomerase (NCT03946358), Ras (NCT00019331), MUC1 (NCT02544880), Arginase-1 (NCT03689192), WT1 (NCT03311334), and IDO (NCT04445064).

## 15. Limitations of Cancer Vaccines

Immunogenic effects of the abovementioned vaccines are limited, primarily because most of tumour antigens are recognised as self-antigens that do not activate antitumour immune reaction. Secondly, there is a high variability of tumour antigen expression among different tumour tissues from different sites due to biological tumour heterogeneity. Additionally, cancerous tissues adopt various immune evasion strategies such as low MHC class I expression and an immunosuppressive tumour microenvironment [1]. These limitations precipitated the emergence of personalized vaccine therapy, which is subject to its own inherent limitations. 

An individual neoplastic lesion comprises thousands of somatic mutations. Prediction of neoantigens that elicit antitumour responses is a daunting task. However, with the advent of omics approaches, prediction of peptide–MHC binding has been highly effective in clinical trials [118], although this accomplishment has been biased towards MHC class I-specific neoantigens. The prediction of MHC class II antigen molecules remains a challenge. The high variance of MHC class II molecules makes selection of an anticipated binding motif difficult.

Another major limitation of cancer vaccine treatment is the time and cost involved in developing personalized vaccines. Currently, development of personalized vaccines takes approximately 3–4 months [118]. For the treatment of metastatic lesions, such a long duration can be detrimental, although patients follow conventional anticancer treatment during the period of personalized vaccine development.

## 16. Preclinical Models for Research: A Special Mention

Given the abovementioned clinical and preclinical trials, the development of preclinical models of human tumour immune environments for assessment of immunotherapies has become imperative. Murine immune-oncology models and PDX models have been utilized to study the efficacy of immunotherapies [119]. However, the rate of successful engraftment is highly variable, and successful development of preclinical models takes a long time. Additionally, ethical hindrances regarding the usage of mouse models is a major limitation [120]. Thus, the use of *Drosophila melanogaster* (*Dmel*) is gaining popularity. The translational relevance of *Dmel* in drug discovery and drug repurposing has been recommended, as many drugs produced comparable effects in both humans and flies [121]. *Dmel* is still not the first choice in drug discovery and drug repurposing endeavours, although fly models can replace murine models for future drug efficacy testing due to their genomic simplicity, ease of use, minimal ethical issues, and cost effectiveness. In fact, carefully considering drug discovery and the development pipeline, *Dmel* can play an indispensable role in personalized immunotherapy [120]. Two clinical trials are worth mentioning in this context. *Dmel*-generated cytotoxic T lymphocytes were used to attach melanoma proteins to leukocytes, for testing the safety and efficacy of modified leukocytes as a treatment for metastatic melanoma (NCT01271907). *Dmel* was also used for the development of personalized cancer therapy in metastatic medullary thyroid or metastatic colon cancer. Mutations identified by DNA and RNA sequencing of individual tumours were screened for tumour drivers and subsequently incorporated into the personal *Dmel* model and studied against a library of FDA-approved drugs. (NCT02363647).

## 17. Conclusions

Immunotherapy has emerged as a ray of hope in the fight against head and neck cancer, as summarized in Table 3.

Despite the use of preclinical cancer models since the 1900s [121], successful modulation of immunity to restrict the progression of cancer has emerged only recently. The advent of cancer vaccines followed the initial successful clinical trials of interleukins and adoptive T-cell transfer to treat HNSCC, culminating in the FDA approving nivolumab and pembrolizumab. Despite these successes, immunotherapies are still challenging. Intratumour heterogeneity of PD-L1 expression is still a concern [122]. Additionally, PD-L1 is difficult to track as a biomarker, as its expression decreases with time in stored pathological samples [123].

The emergence of personalized therapy using preclinical models such as *Dmel* and the prediction of MHC class I antigens via an omics approach have greatly contributed to the recent success of clinical trials. Immunotherapy-related adverse effects are common, but they are better tolerated than conventional chemotherapy. A multidisciplinary team approach comprising bioinformatics, genetic engineering, clinical oncology, immunology, pharmaceutics, and molecular biology to develop personalized immunotherapy is another great accomplishment. These strategies highlight the intrinsic relation between fundamental science research and clinical practice. Finally, through this review, we have highlighted the intrinsic connection between basic science research and clinical practice, setting the paradigm for future research and development in the field of HNSCC.

## Figures and Tables

**Table 1:** FDA-approved immune checkpoint blockers.

Type of Cancer	Immune Checkpoint Blocker
Squamous cell head and neck carcinoma	Nivolumab or pembrolizumab
Malignant melanoma	Ipilimumab, nivolumab, pembrolizumab
Merkel cell carcinoma	Avelumab, pembrolizumab
Hepatocellular carcinoma	Nivolumab, pembrolizumab
Cutaneous squamous cell carcinoma	Cemiplimab
Advanced renal carcinoma	Nivolumab or ipilimumab
Colorectal cancer	Nivolumab or ipilimumab
Cervical cancer	Pembrolizumab
Small cell lung cancer	Atezolizumab, nivolumab
Non-small cell lung cancer	Durvalumab, pembrolizumab, atezolizumab, nivolumab
Triple-negative breast cancer	Atezolizumab
Gastric carcinoma	Pembrolizumab
Hodgkin lymphoma	Pembrolizumab
Primary mediastinal large B-cell lymphoma	Pembrolizumab
Metastatic urothelial cancer	Durvalumab, pembrolizumab, atezolizumab, nivolumab, avelumab

**Table 2:** Interleukins and cancer.

Interleukin	Effect of Interleukins in Cancer	Reference
IL-1	VEGF angiogenesis; MMP metastasis; COX-2, iNOS, PGE2, and IL-17 induction	[73]
IL-2	A growth factor for T_effector_ cells; promotes antitumour immunity	[74]
IL-3	Activation of c-MYC, c-Fos, and c-FMS; protumour effect; helps in anti-apoptosis, and proliferation; affects osteoclast/osteoblast formation; aids in survival of malignant haematopoietic cells	[75]
IL-4	Angiogenesis; increases clonogenic potential of stem cells, as well as AKT, p44/42 MAPK, NF-κB, and JAK/STAT6 pathways; immunosuppression	[76]
IL-5	Facilitates metastasis; overexpression indicates poor prognosis	[77]
IL-6	Signalling of cell survival, proliferation, metastasis, and angiogenesis, with profound effects on cancer	[78]
IL-7	Antiapoptotic and proliferation	[79]
IL-8	Assists in progression of cancer; affects gene expression; modulates translation and post-translational modifications of proteins, affecting cytoskeletal organisation, tumour immune resistance, and angiogenesis; accentuates proliferation signals and attracts myeloid cells to provide growth factors	[80]
IL-9	Induces T_H_9 cells, STAT6, IRF4, and PU.1; regulates intestinal barrier function via regulation of claudin-2.	[81]
IL-10	Plays a role in the regulation of various subsets of CD4^+^ T cells; in combination with IL-4 and IL-2 helps in proliferation of CD8^+^ T cells; IL-10-treated DCs induce an anergic state in CD4^+^ and CD8^+^ T cells; IL-10/STAT3 mediates anti-inflammatory response and promotes survival of B cells, MHC class II suppression, with both tumour promotion and inhibitory roles	[82]
IL-11	Aids in the progression of cancer by influencing the IL-11/STAT3 axis	[83]
IL-12	Tumour suppression; increases antiangiogenesis and antiapoptotic factors; increases IL-2R and IFNγ.	[84]
IL-13	Evasion and metastasis; suppresses CTL responses against cancer; facilitates growth of cancer.	[85]
IL-14	B-cell proliferation	[86]
IL-15	Antitumour immunity	[87]
IL-16	Chemoattractant; growth factor	[88]
IL-17	Angiogenesis, metastasis, and lipocalin	[89]
IL-18	Modulates T_H_1 differentiation, pro-inflammatory activity, and NK-mediated cytotoxicity; upregulates IFN-γ, TNF-α, IL-1β, and IL-8; activates MAPK and STAT3	[90]
IL-19	Induces JNK, ERK, Akt, NF-κB, STAT1, and STAT3; upregulates MMP2 and MMP9; promotes proliferation of oral squamous cell carcinoma	[91]
IL-20	Increases migration and proliferation of cancers through activation of bFGF, VEGF, STAT3, ERK, JNK, and Bcl-xL	[92]
IL-21	Activates the JAK/STAT, PI3K, and MAPK pathways; enhances cytotoxic activity of NK and NKT cells; induces DC apoptosis	[93]
IL-22	Upregulates proliferative pathways such as JAK/STAT, PI3K/Akt, NF-κB, MAPK, and mTOR	[94]
IL-23	IL-23 and TGFβ suppress metastasis in pancreatic cancer; IL-23 and IL-1β drive T_H_17 differentiation and promote growth and progression of oral squamous cell carcinoma	[95]
IL-24	Tumour suppressor; suppression of glial inflammatory response	[96]
IL-26	Proinflammatory and prosurvival activities by regulating the balance of STAT1 and STAT3 activity	[97]
IL-27	Anticancer effect; reduction in VEGF and a series of angiopoietins; inhibits T_reg_; downregulates MMP9; inhibits COX-2 and PGE2	[98]
IL-28	IL-28A promotes upregulation of MHC I and suppression of Th 17 and Th 2 responses	[99]
IL-30	Binds to gp130 and blocks pathways affected by IL-6 and IL-27. Binding to gp130 activates STAT1/STAT3 and promotes cancer progression; promotes BRCA cell migration and invasiveness	[100]
IL-31	The IL-31/IL-31R axis recruits OSMR, activating MAPK and PI3K/Akt	[101]
IL-32	Overexpression in HNSCC induces TNFα, IL-6, and IL-1β, as well as macrophage inflammatory protein-2 (MIP-2)	[102]
IL-33	IL-33/ST2 activates T_H_2-polarized cells, leading to the induction of IL-4, IL-10, and IL-13; generates immature DCs, which induce T_reg_	[103]
IL-34	High coexpression of IL-34 and M-CSF correlates with poor prognosis in lung cancer patients; promotes tumour progression and metastasis via induction of angiogenesis and macrophage recruitment	[104]
IL-35	CD11b (+) Gr1(+) myeloid cells promote cancer; cell cycle arrest in G1 phase; apoptosis due to TNFα; IFNγ-mediated upregulation of Fas ligand with simultaneous downregulation of cyclin D1, survivin, and Bcl-2	[105]
IL-36	IL-36α suppresses proliferation of cancer cells; exerts proinflammatory activity through MAPK and NF-κB	[106]
IL-37	Inhibits T-cell and DC activation; promotes IL-10 and IL-16; regulates STAT3; promotes T_reg_; inhibits Smad3/TGFβ; inhibits proliferation and invasion of cancer cells	[107]
IL-38	Inhibits IL-8, IL-17, and IL-22; overexpression is related to poor prognosis	[108]

**Table 3:** Summary of the progress of immunotherapy in head and neck cancer.

Treatment Option	Progress
Adoptive T-cell transfer	CD70/CART cell therapy not appropriate for HNSCC treatment; TIL (LN-145) undergoing clinical trial (NCT03083873)/
Immune checkpoint therapy	Pembrolizumab (PD1 inhibitor) and nivolumab (monoclonal antibody against PD1) approved by the FDA for the treatment of HNSCC; Cetuximab and Ipilimumab not FDA-approved for the treatment of HNSCC.
Interleukin therapy	IL-36γ in combination with IL-23 and OX40 ligand is currently undergoing a phase I clinical trial; ALKS4230) in combination with pembrolizumab is under clinical trial (NCT04144517).
Cancer vaccines	MEDI0457 in combination with durvalumab is under clinical trial (NCT03162224); Several non-viral antigen vaccines are under clinical trial.
Personalized therapy	*Drosophila melanogaster* is currently used by various researchers for the treatment of head and neck cancers
